# Electromyographic activity of the jaw muscles and mandibular kinematics in young adults with theoretically ideal dental occlusion: Reference values

**DOI:** 10.4317/medoral.21631

**Published:** 2017-04-08

**Authors:** Bárbara Campillo, Conchita Martín, Juan-Carlos Palma, Aler-Daniel Fuentes, José-Antonio Alarcón

**Affiliations:** 1 Department of Stomatology IV, School of Dentistry, Complutense University of Madrid, Madrid, Spain; 2 Institute for Research in Dental Sciences, Faculty of Dentistry and Oral Physiology Laboratory, Biomedical Sciences Institute, Faculty of Medicine, University of Chile, Santiago, Chile; 3 Department of Stomatology, School of Dentistry, University of Granada, Granada, Spain

## Abstract

**Background:**

A necessary step to use neuromuscular analysis as diagnostic tool is to establish normal reference values for the physiological range in a healthy population. Surface electromyographic (sEMG) activity of the jaw muscles and mandibular kinematics were measured in young adults with theoretically ideal dental occlusion to determine normal reference values during different tasks. Differences between the sexes were evaluated.

**Material and Methods:**

Forty young adults (20 men, 20 women; mean age 22.8 ± 3.9 years) with theoretically ideal dental occlusion were selected using very restrictive criteria. sEMG activity of the anterior temporalis (AT), posterior temporalis, masseter (MA), and suprahyoid muscles were evaluated in the rest position and during swallowing, mastication, and clenching. Mandibular kinematics in the rest position and during maximum excursions were assessed. Asymmetry, activity, and torque indices and MA/AT ratios were calculated.

**Results:**

For all muscles, sEMG values were 1.01-3.57 µV at rest, 3.50-10.85 µV during swallowing, and 41.04-86.59 µV during mastication. During clenching, values were 230.08-243.55 µV for the AT and MA muscles. Mean total asymmetry, activity, and torque indices at rest were 20.34 %, -15.04 %, and 19.02 %, respectively; during clenching, these values were 6.14 %, -2.62 %, and 4.46 %. MA/AT ratios were near 1. Kinematic measurements during lateral excursion, protrusive and maximum opening were 7.54, 8.44, and 37.38 mm respectively; lateral mandibular shift was 1.41 mm; free way and lateral displacement at rest were 1.40 and 0.26 mm. Right MA activity during mastication and clenching was higher in men than women.

**Conclusions:**

Reference values for sEMG activity and mandibular kinematics were determined. Some muscular asymmetry and torque were observed.

** Key words:**Electromyography, masticatory muscles, kinesiography, jaw movements, normal dental occlusion, sexual dimorphism.

## Introduction

Neuromuscular analyses, including surface electromyography (sEMG) of the masticatory muscles and kinematics of the mandible, have been widely employed in clinical practice and research. These techniques can quantify the amount of disharmony in pathological conditions as well as the outcome of given therapies. To properly employ these methods, a necessary first step is to establish normal reference values for the physiological range in a healthy population with normal occlusion. While several attempts have been made to establish these values, well-defined data and well-controlled clinical EMG studies remain scarce.

The main limitation of previous studies is insufficient control of factors that can influence jaw muscle sEMG activity and mandibular kinematics during sample selection. sEMG activity of the masticatory muscles has been found to vary with skeletal sagittal and vertical facial patterns (1,2), as well as with skeletal asymmetries (3) and posterior cross-bite (4). Masticatory muscle activity can also be affected by occlusal interference. Ferrario *et al.* (5) found sEMG alteration in the jaw and neck muscles when an occlusal interference was introduced in healthy subjects. Other studies have concluded that mandibular kinematics can be influenced by skeletal sagittal (skeletal class) and vertical (6) patterns, as well as by age (7) and gender (7,8).

Therefore, it is crucial to take all of these variables into account when recruiting subjects to determine normal reference values. To the best of our knowledge, no single study in subjects with healthy normo-occlusion has used inclusion criteria covering all of these variables. To name some specific studies, Ferrario *et al.* (9) examined 92 healthy young people, but used the soft criteria of absence of moderate or severe clinical mandibular disorders (no temporomandibular jointsounds, no tenderness on palpation of the temporomandibular joint or of the masticatory muscles, no painful limitation of mandibular movement), which could have allowed inclusion of patients with some clinical symptoms of mandibular disorders (10). De Felício *et al.* (11) only analyzed 20 normal subjects, and recognized that “even in this normal group, TMD signs and symptoms were reported by a few subjects.” Even the recent study by Wieczorek and Loster (10) included Class I, II, and III patients in their control group of non-orthodontically treated participants. These circumstances could invalidate the data obtained in these studies as normal reference values. Only a group selected using very restrictive criteria, and that meets all requirements of theoretically ideal dental occlusion, should be used to determine normal reference values. The components of theoretically ideal dental occlusion include Angle Class I occlusion; overjet and overbite within normal range; proper occlusal vertical dimension with properly related vertical, transverse, and antero-posterior relationships; centered midlines; and regular arch form, with no crowding, protrusive incisive guidance, laterotrusive canine guidance, or group function (12).

The aim of the present study was to evaluate a sample of young adults with narrowly defined theoretically ideal dental occlusion to determine normal reference values for sEMG activity of the masticatory muscles; their indices for muscular asymmetry, activity, and torque; the ratio of masseter (MA) to anterior temporalis (AT) activity; and mandibular kinematics, during different functional tests. In addition, we tested the hypothesis that gender influences both masticatory muscle sEMG and mandibular kinematics.

## Material and Methods

- Subjects

Forty healthy subjects (20 men, 20 women) aged 20 to 30 years (mean 22.8 ± 3.9 years) with theoretically ideal dental occlusion were recruited from among approximately 1200 students at the Complutense University of Madrid, Spain, according to very restrictive inclusion and exclusion criteria.

Participants were selected based on anamnesis and clinical exam. All subjects underwent an orthodontic and temporomandibular joint functional examination, performed by the same trained orthodontist (the first author), who was calibrated to a gold standard (for details, see International Consortium for RCD/TMD-based Research, 2006) (13).

The inclusion criteria were: age between 20 and 30 years; Caucasian origin; complete natural permanent dentition (excluding third molars); skeletal Class I (based on ANB angle, convexity, and Wits appraisal, as measured on lateral cephalogram); mesofacial growth pattern (according to the Frankfort horizontal-to-mandibular plane angle on lateral cephalogram); Angle Class I occlusion; overjet and overbite of 2 ± 1 mm; centered midlines; regular arch form with no crowding, protrusive incisal guidance, laterotrusive canine guidance, or group function; nasal breathing; normal-mature swallowing; and lip competence.

The exclusion criteria were: any TMD diagnosis based on the Research Diagnostic Criteria for Temporomandibular Disorders (14); history of neuromuscular disease or disease affecting neuromuscular performance; headaches and/or other neurological disorders; systemic and/or localized maxillofacial diseases; craniofacial anomalies; history of cervical, head, or dental trauma; current or past orofacial, myofunctional, or TMD treatment; bruxism (diagnosed based on parafunctional facets and/or self-reported history of tooth clenching and/or grinding); parafunctional habits; habitual use of chewing gum; presence of dental pain, periodontal disease, gingival recession, fractured teeth, dental prostheses, occlusal wear, or large restorations that included an incisal edge or one or more cusps; absence of one or more teeth with the exception of the third molars; supernumerary teeth or tooth agenesis (confirmed with panoramic X-ray); presence of skeletal asymmetries (confirmed on frontal and Hirtz radiographs); anterior or posterior cross-bite; presence of diastemas; slide from retruded contact position to intercuspal greater than 2 mm; mediotrusive and/or protrusive occlusal interference; recent or current orthodontic treatment; use of any medication that could affect muscle activity, such as antihistamines, sedatives, central nervous system depressors, and psychiatric drugs; speech therapy; otorhinolaryngological treatments; and botulinum toxin therapy.

Participants volunteered for the study after a detailed explanation of the experimental protocol and agreed to participate by signing an Ethics Committee-approved informed consent. None of the procedures in this study were dangerous or painful. The study was approved by the local ethic committee (B-05/327 Comite Etico de Investigación Clinica, Hospital Clinico San Carlos).

- Electromyography measurement

The study was performed with an EM2® electromyograph (K6-I Diagnostic System®, Myotronics-Noromed, Kent, WA, USA), with eight channels and a frequency bandwidth response of 45-430 Hz per channel, that allows four pairs of muscles to be simultaneously tested. Disposable silver/silver chloride bipolar surface electrodes (Duo-Trode, Myotronics-Noromed, Kent, WA, USA) were positioned on the muscle bellies parallel to the muscle fibers (inter-electrode distance, 21 ± 1 mm), according to a previously described protocol (4).

Experimental electromyographic procedures

Simultaneous bilateral sEMG activity (in µV) of the AT, posterior temporalis (PT), MA, and suprahyoid (SH) muscles was recorded at the mandibular rest position, during swallowing, and during mastication. sEMG activity of the bilateral AT and MA was also recorded during maximal voluntary clenching (MVC) in maximal intercuspation.

During the experiment, subjects were seated in an upright position with the Frankfort plane parallel to the floor, in a quiet, dark, and comfortable environment. Verbal instructions were given to the participants before starting to avoid any stressful situation. Trial tests were permitted before the definitive recording. Irregular or spurious tracings were excluded for all tasks. One calibrated examiner (MCM) performed all EMG measurements.

First, sEMG activity was recorded in the mandibular rest position. Patients were asked to refrain from swallowing during the recording phase and to keep their eyes closed. Three consecutive 15-sec recordings were made at 2-min intervals; the mean value was considered the resting sEMG activity. To determine sEMG activity during MVC, subjects were encouraged to clench as hard as possible in maximal intercuspation. Three 3-sec MVC trials were recorded at 2-min intervals. The highest sEMG activity measured was considered the sEMG activity during MVC. For swallowing, sEMG was recorded as follows: subjects were instructed to take a mouthful of water and to hold their jaws in the rest position. They were then instructed to swallow the water and to hold their jaws in the rest position again after swallowing. The peak activity (maximum amplitude) was measured; a 1-min rest period was allowed between swallows. Last, sEMG was recorded during mastication. The operator asked subjects to eat potato chips; no further instructions were given. Average sEMG activity of the last 10 sec of mastication was recorded (4).

Indices of asymmetry, activity, and torque were calculated for each muscle in the mandibular rest position and during MVC to determine muscular balance. All indices could vary from -100 % to +100 %, and were calculated as follows (15):

Total asymmetry index = (MAright + ATright− MAleft− ATleft)/(MAright + ATright + MAleft + ATleft) %

Partial asymmetry index = (right muscle − left muscle)/(right muscle + left muscle) %

Activity index = (MAright + MAleft− ATright− ATleft)/(MAright + MAleft + ATright + ATleft) %

Torque index = (ATright + MAleft− ATleft− MAright)/(ATright + MAleft + ATleft + MAright) %

For the total and partial asymmetry indices, negative numbers indicate the predominance of the left muscle, while positive numbers indicate right-muscle predominance. A zero value reflects similar muscle activity on both sides. For the activity index, negative numbers indicate prevalence of AT activity, whereas positive numbers indicate prevalence of MA activity. For the torque index, positive values indicate a stronger right side couple; negative values, a stronger left side couple.

In addition, the MA/AT ratio during MVC was also recorded to evaluate the relationship between MA and AT muscle activity in each subject. A ratio larger than 1 indicated greater MA than AT muscle activity; a ratio less than 1 indicated greater AT than MA activity (16).

To assess the reproducibility of EMG data, five subjects underwent four trials over a 4-day period.

- Kinematic measurements

Mandibular movements and positions were recorded using a kinesiograph computer system (K6-I Diagnostic System®, Myotronics-Noromed, Kent, WA, USA), according to previously described protocols (17). Kinematic measurements (in mm) were recorded during maximum excursions, including maximum vertical opening, lateral mandibular shift (from maximum vertical opening to maximum intercuspation), protrusion, and right and left lateral excursions, and at the rest position, including vertical freeway space and lateral displacement (from rest position to maximum intercuspation).

One calibrated examiner (MCM) performed all kinesiographic measurements. The reproducibility of the kinesiographic records was tested by comparing the results of two consecutive measurements of 10 randomly selected subjects, according to a previously published method (17).

- Statistical analysis

The analysis of variance (ANOVA) for repeated measures test was used to test sEMG measurement reproducibility. Kinesi-ographic data reproducibility was tested with the paired t-test. Descriptive statistics (mean values and 95 % confidence intervals) were calculated for sEMG activity, indices, MA/AT ratios, and measurements of mandibular kinematics. After establishing the normal distribution of variables with the Kolmogorov–Smirnov and Shapiro–Wilk tests, data from men and women were compared with Student’s t-test for independent samples or the Mann-Whitney U-test.

The data were analyzed with SPSS 15.0 software (SPSS Inc., Chicago, IL, USA). Values of *p* ≤0.05 were considered statistically significant.

## Results

No statistically significant differences were found among repeated recordings in the sEMG reproducibility test (*p*>0.05; repeated measures ANOVA). A paired t-test found no systematic differences between the first and second kinesiographic recordings (*p*>0.05).

 Table 1 shows bilateral sEMG activity in the AT, MA, PT, and SH muscles during all tasks in the total test population and according to participant sex. Higher values were found in the PT in the mandibular rest position and in the SH muscles during swallowing in all groups. During mastication, the lowest sEMG activity was found in the SH muscles in all groups, followed by the PT muscles; no clear predominance of activity between the AT and MA muscles was found. During MVC, the mean values were higher in the AT muscles, both in the whole sample population and among women. However, higher values were found in the MA muscles among men. Comparisons according to sex show no significant differences, except in the right MA muscle area during mastication and MVC, which had a higher sEMG activity in men than in women.

**Table 1:**
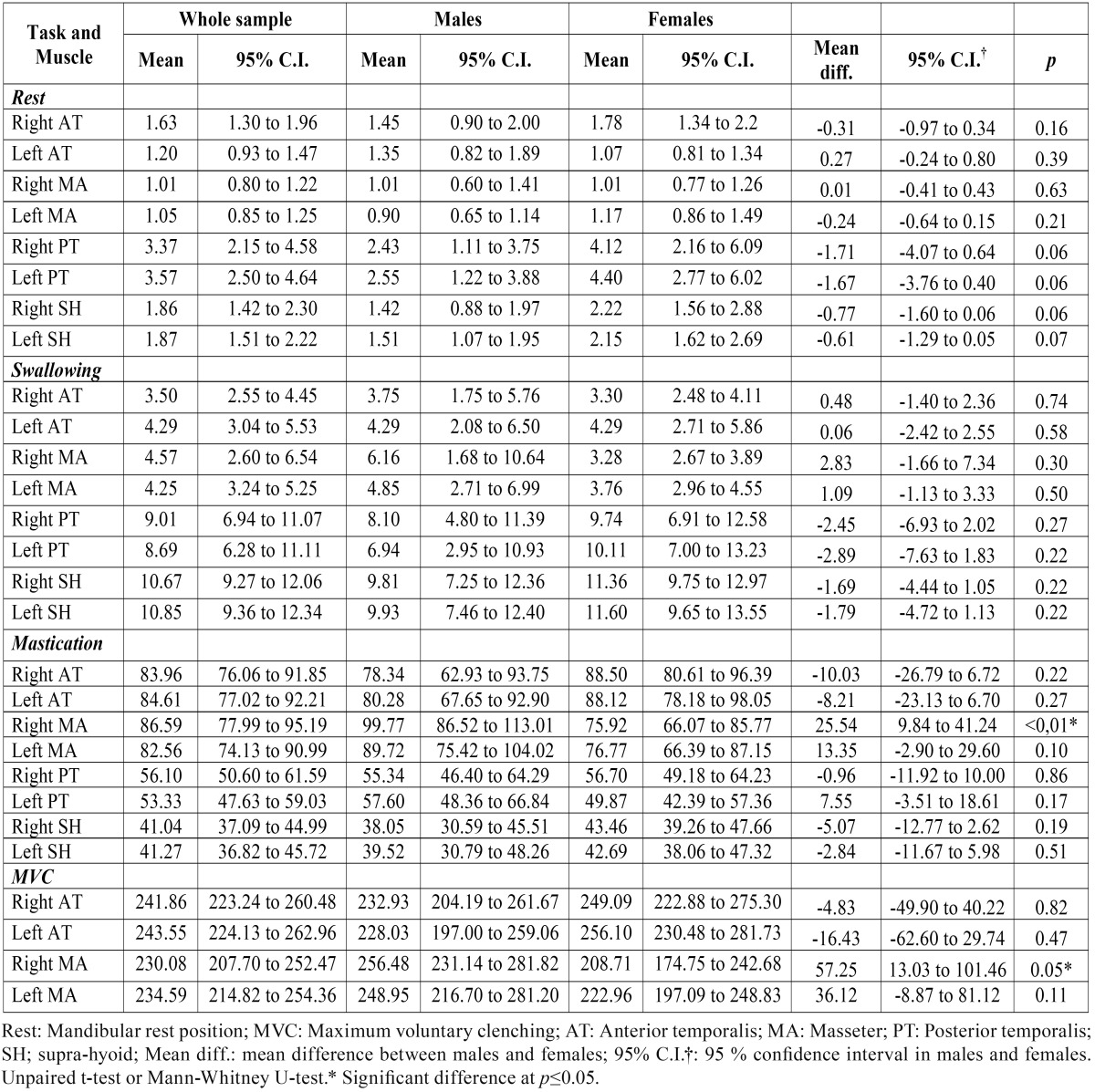
Descriptive statistics (mean values, 95% confidence intervals) and comparisons of sEMG activity (µV) at mandibular rest position and during swallowing, mastication and maximum voluntary clenching between male and female groups.

 Table 2 shows the values for asymmetry, activity, and torque indices (%). In the mandibular rest position, the asymmetry index ranged from 20.10 % to 30.15 %, the activity index ranged from -17.41 % and -13.12 %, and the torque index ranged from 15.67 % and 19.16 % in all groups. During MVC, the asymmetry index ranged from 5.67 % to 12.09 %, the activity index ranged from -8.87 % to 5.10 %, and the torque index ranged from 3.43 % to 5.30 %. Finally, MA/AT ratios ranged from 0.82 to 1.13 in all groups. There were significant differences between indices of men versus women only during MVC. The MA asymmetry index was higher in women than in men; however, in both groups this value was positive. The activity index was higher in men than in women. In men the value was positive (5.10 %), whereas in women this value was negative (-8.87 %). Right and left MA/AT ratios were significantly higher in men than in women.

**Table 2:**
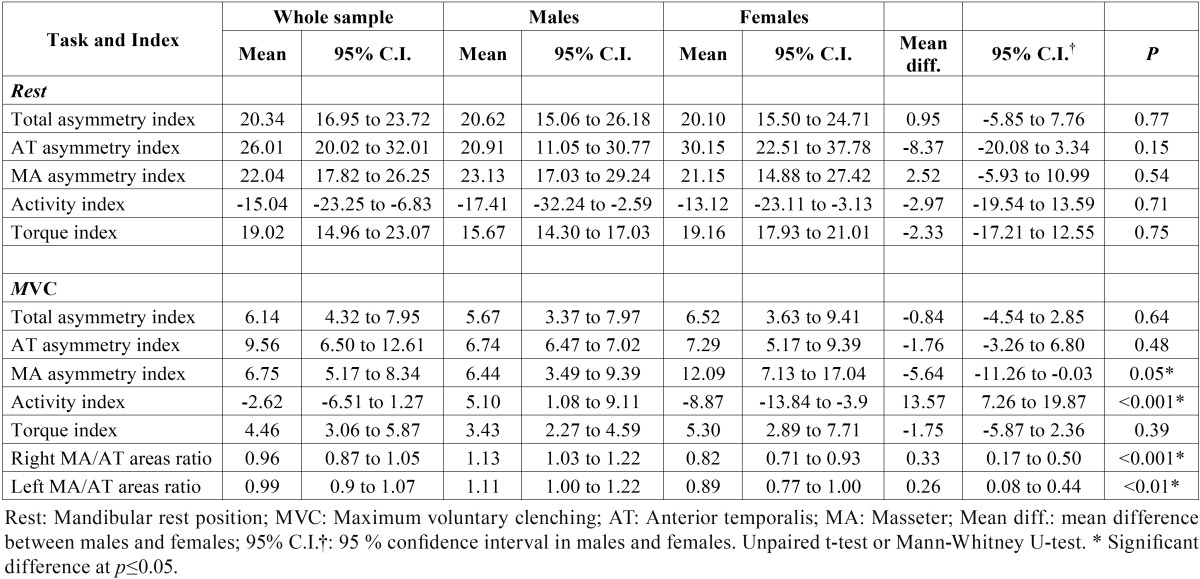
Descriptive statistics (mean values, 95% confidence intervals) and comparisons of asymmetry, activity, and torque indices (%) at mandibular rest position (mean sEMG activity) and during maximum voluntary clenching (peak sEMG activity), and right and left masseter/anterior temporalis ratios during maximum voluntary clenching, between male and female groups.

 Table 3 shows kinematic measurements (mm) during maximum excursion and in the mandibular rest position. Higher values were found during maximum vertical opening, and lower maximum excursion values were found in lateral mandibular shift. Lateral excursion values (right and left) and protrusion were similar. The lowest values were found for lateral displacement and vertical freeway space. There were no significant differences in kinematic measurements in men versus women during maximum excursion and in the mandibular rest position.

**Table 3:**
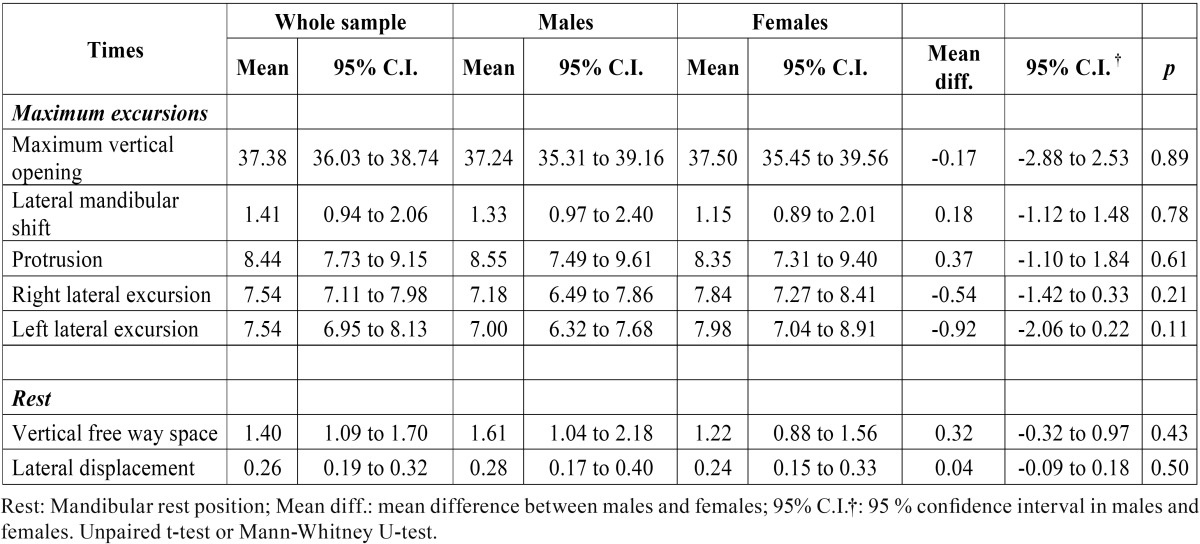
Descriptive statistics (mean values, 95% confidence intervals) of kinematic measurements of the mandible (mm) and comparisons during maximum excursions and at mandibular rest position between male and female groups.

## Discussion

sEMG activity of the jaw muscles and kinematic measurements of mandibular position and movements were registered in subjects with theoretically ideal dental occlusion, to obtain normal reference values during different tasks. Differences between the sexes were also evaluated. Normal ranges of sEMG activity variability in the rest position and during swallowing, mastication, and clenching in maximal intercuspation were determined. Asymmetry, activity, and torque indices, and MA/AT ratios were also obtained. Reference kinematic data for the mandible in the rest position (vertical freeway space and lateral displacement), and during maximum excursions (maximum vertical opening, lateral mandibular shift, protrusion, and right and left lateral excursion) were also determined. A certain degree of muscular asymmetry and torque effect were found. For sEMG activity, the null hypothesis of no difference between the sexes was rejected, because right MA activity during mastication and clenching was greater in men than in women. The null hypothesis was accepted for mandibular kinematics, because no significant differences were found between the sexes.

The normal reference values of sEMG masticatory muscles and kinematic of the mandible reported in our study are useful in many fields of Dentistry (i.e. orthodontics, temporomandibular disorders, craniofacial pain, prosthodontics, maxilla-facial surgery, etc.), and could have special relevance in the diagnostic and treatment monotorization of bruxism and temporomandibular disorders, problems that are increasing lately related with stress and accelerated way of life. Our results could also have applicability in other areas of Medicine, for example in Neurosciences, Physiology, Rehabilitation or Physiotherapy, to name but a few, as well as in research.

sEMG is a currently accepted, noninvasive, reproducible technique that can be used to evaluate muscular function, when appropriate protocols are applied (9). Similarly, kinesiographic measurements were performed following standardized, well-designed protocols (18). Several studies show that tests made using sEMG do not differ significantly from intramuscular tests and are easily reproducible when performed with properly standardized protocols (19-21).

In the present study, mean sEMG activity values in the mandibular rest position were similar to those found by Scopel *et al.* (22) and Cha *et al.* (1), while other studies have reported higher values (2,9). These differences can be attributed to variations in sample selection criteria. sEMG activity was highest in the PT muscle area, followed by the SH, AT, and MA areas, a finding that is in line with other investigations (1,2,9,22). This result can be explained by the function of each muscle and the relationship between function and number of muscle spindles. The temporal muscle had the largest number of muscle spindles of the analyzed jaw-closing muscles, with more spindles in the posterior than in the anterior portion; the MA muscle had the next most spindles of the jaw-closing muscles. Thus, the jaw-closing spindles should have a strong proprioceptive impact and, PT and AT muscles would play a principal role as postural muscles in the mandibular rest position (9).

As expected, higher sEMG activity was found in the SH muscle area during swallowing, as reported in other studies (23). This finding can be attributed to the important function of the SH muscles in elevating the hyolaryngeal complex during swallowing .

It is difficult to compare our results for mastication with other investigations because of differences in experimental methodology. Moreno *et al.* (23) found lower sEMG values, but their study included subjects with malocclusion (e.g., Angle classes II and III, posterior cross-bite, anterior open bite). The function of the jaw muscles during the chewing cycle has been described as follows: the MA provides power during jaw closing, the AT cooperates in this work, and the PT stabilizes the mandible in the horizontal plane and also is involved in lateral and retrusive jaw movements . In our study, the sEMG activity of the right MA was higher in men than in women, while the left MA did not show significant differences by sex. This asymmetric difference could be related to a right-sided masticatory predominance in men.

During MVC, both studied muscles (AT and MA) showed sEMG values over 200 µV. These results are in agreement with those of Cooper (24), who named a minimum of 125 µV as a normal value for this task in the AT and MA muscles. Only the right MA showed significantly higher values in men (mean, 256 µV) than in women (mean, 208 µV), reflecting predominant sEMG activity in the right MA in men, which could also be explained by a right-sided masticatory predominance. Ferrario *et al.* (9) and Cha *et al.* (1) found higher EMG activity in men than women in both right and left MA muscles. The difference in study findings could also be attributed to the smaller sample size of our study. It should be noted that there is scientific evidence that shows that records of sEMG of the masticatory muscles in MVC may be distorted by voluntary and involuntary activity of adjacent facial, head and cervical muscles. Abbink et al. suggest that the results obtained by sEMG should not be considered the most accurate and precise. Therefore, the results during MVC must be consider carefully (25).

Slightly asymmetrical muscle activity in normal healthy subjects is a general rule in the literature (9,11,15,22,26). In our study, a certain degree of muscular asymmetry (total and partial) was found in the mandibular rest position and, to a lesser extent, during clenching. We agree with Ferrario *et al.* (26) that even in highly selected subjects whose occlusion is good from a morphologic perspective, the presence of a prevalent side or couple seems to be an intrinsic asymmetric characteristic of occlusion, independent of biological noise. In our study, women had a higher MA asymmetry index during clenching than men; other studies did not find a sex difference (9,26). Asymmetry indices were lower during clenching than in the rest position, indicating that asymmetry is higher when muscle activity is lower (3). The present study also found that total asymmetry indices were lower than partial asymmetry indices, in both the rest position and during clenching, and in both sexes. These findings are in agreement with other studies (9,22), and can be explained by an apparent compensation factor between the two muscles, because AT and MA asymmetries had opposite signs.

Activity indices showed an AT predominance over MA (approximately 15%) in the rest position, with no differences between the sexes. Similar results have been reported previously (9,15,22). These findings indicate that in healthy young adults with normal occlusion, jaw posture is controlled more by the AT than the MA muscles. Activity indices were lower during clenching than in the rest position, indicating balanced contribution of both the AT and MA muscles, as described in other studies (11). Significant differences were found in activity indices of men (mean 5.10 %) versus women (mean -8.87 %). Although the results reflect a balanced contribution of AT and MA muscles in both sexes, MA predominates over TA in men, whereas in women the opposite is true. Similar results have been found in other studies (9,10,15). Similar to other studies of normo-occlusive subjects (9,11,22,26), torque indices in our participants showed a certain degree of lateral jaw torque effect, generated by AT activity on one side and contralateral MA activity. Ferrario *et al.* (9) found a torque index of 11.59 % at rest and 9.47 % during clenching. Our indices were higher at rest (19.02 %) and lower during clenching (4.46 %); however, in both studies, torque indices were lower during clenching (more muscular activity) than at rest (lower muscular activity). One explanation for these findings could be that the torque effect in the horizontal plane is usually counterbalanced by forces generated from other anatomical structures. At rest, this balance is produced by other muscles, such as the AT and the lateral and medial pterygoid muscles, while during maximum clenching the tooth action is added, preventing lateral jaw deviation (9,22). A similar explanation could be applied to the asym-metry index findings. As in the study by Ferrario *et al.* (9), no sex differences were found in torque indices.

In our investigation, the mean MA/AT ratios were close to 1 in the total sample, in male and female groups, and on both sides. These results indicate that the sEMG activity of the AT and MA is balanced during MVC. In line with our activity indices results, right and left MA/AT ratios showed significant differences between the sexes. In men, the MA showed higher activity than the AT; in women the opposite was true.

Kinematic measurements of mandibular movements revealed no differences between the sexes in maximum vertical opening or lateral mandibular shift. Ferrario *et al.* (27) found similar values in healthy subjects, without sex differences (mean of 37 mm in males and 36.18 in females). In contrast, Lewis *et al.* (8) found higher values in men (mean, 45.32 mm) than in women (mean, 38.75) in maximum vertical jaw opening. Others authors have also found higher maximum vertical opening values in males than in females, using both kinematic records (7,28) and clinical measurement with calipers (29). Mandible size and body height, which are related to sexual dimorphism, could play a role in these sex differences in maximum vertical opening. Lewis *et al.* (8) found no relationship between maximum opening and mandible size, while Gallagher *et al.*(29) found no relationship with body height. However, Mapelli *et al.* (30) found higher mandibular opening values in males than in females, a difference that disappeared when mandibular radius was applied as a correction factor. More specific studies are needed to confirm this hypothesis.

Protrusion values were similar between the sexes, with a mean value of 8.44 mm for the total sample. This finding is in line with that of Hirsch *et al.* (7), who reported a mean protrusion of 8.3 mm in males and 8.0 mm in females, although their sample included 10–17-year-old children and adolescents. In contrast, Sierpinska *et al.* (28) found higher values in men (mean, 7.12 mm) than in women (mean, 5.79 mm) in a sample of normo-occlusive 18-21-year-olds.

The mean lateral excursion of 7.54 mm found in our sample is close to the minimum normal value of 7 mm proposed by Cooper (24) in his “parameters of an optimal physiological state of the masticatory system.” Others studies (7,28) have found higher values in both the right and left sides than our study did; however, as in our study, no difference was found between the sexes. Different methodologies and sample selection criteria, including age, could account for these disagreements.

Finally, in the rest position, a mean freeway space of 1.40 mm and a mean lateral displacement of 0.26 mm were registered in our total sample. These values are in the normal range proposed as the optimal physiological state of the masticatory system by Cooper (24) (between 0.83 mm and 2.34 mm for freeway space, and a mean of 0.07 ± 0.28 mm for lateral displacement). In our study, no significant differences in freeway space or lateral displacement were found between men and women. Michelotti *et al.* (6) also found no differences between the sexes, with similar results to those of our study. However, Ferrario *et al.* (27) found higher values for vertical freeway space in males (mean, 1.94 mm) than in females (mean, 1.06 mm).

s-EMG is a useful tool in research that provides a better understanding of mandibular function. However, it has limited value as a diagnostic and monitoring tool in the management of TMD, because of the multifactorial pathophysiology of these clinical entities. It would be advisable to design and repeat already performed experiments with s-EMG using needle EMG to corroborate the conclusions reached by a more accurate technique.

To summarize, normal values for sEMG jaw muscle activity and kinematic data of the mandible during different tasks were determined for young adults with theoretically ideal dental occlusion. A certain degree of muscular asymmetry and torque effect were shown.

Right MA activity during mastication and clenching was higher in men than in women, while no significant sex differences were found in mandibular kinematics.
